# Acceptability of Overdose Prevention Sites in the Business Community in Baltimore, Maryland

**DOI:** 10.1007/s11524-022-00647-1

**Published:** 2022-05-24

**Authors:** Susan G. Sherman, Saba Rouhani, Rebecca Hamilton White, Noelle Weicker, Miles Morris, Kristin Schneider, Ju Nyeong Park, Colleen Barry

**Affiliations:** 1 Department of Health, Behavior and Society, Johns Hopkins Bloomberg School of Public Health, Baltimore, MD USA; 2 Alpert Medical School, Brown University Division of General Internal Medicine, Providence, RI USA; 3 Jeb E. Brooks School of Public Policy, Cornell University, Ithica, NY USA

**Keywords:** Overdose prevention sites, Opioid epidemic, Harm reduction, Business support of harm reduction

## Abstract

Intervetions are urgently needed to reduce the trajectory of the US opioid overdose epidemic, yet implementation is often hampered by resistance or opposition from key community stakeholders. While businesses are economically and physically impacted by the opioid epidemic, they are rarely engaged in efforts to reduce its impact. The establishment of overdose prevention sites (OPS) is being discussed throughout many US jurisdictions with limited attention to the potential positive role of businesses in that process. We surveyed business owners and employees of businesses located in neighborhoods with concentrated drug markets. The study’s primary aim was to examine their attitudes to locally-placed OPS. An iterative, two-phase sampling strategy was used to identify recruitment zones. In person (December 2019–March 2020) and telephone-based (April–July 2020) surveys were administered to distinct business owners and employees (*N* = 149). Sixty-five percent of participants supported OPS in their neighborhood and 47% had recently witnessed an overdose in or around their workplace. While 70% had heard of naloxone, and 38% reported having it on the premises. Correlates of supporting an OPS locally included living in the same neighborhood as work (adjusted odds ratio (aOR) 1.99, 95% confidence intervals (CI): 1.30–3.05); having a more positive attitude towards people who use drugs (*aOR* 1.33, 95% *CI*: 1.13–1.58); and having recently seen an overdose in/around the workplace (*aOR* 2.86, 95% *CI*: 1.11–7.32). Lack of support being an owner (*aOR* 0.35, 95% *CI*: 0.15–0.83). These data indicate the extent to which businesses are directly impacted by the opioid epidemic and the power of personal experience in shaping OPS support in advocacy efforts.

## Introduction

The USA reported an unprecedented number of overdose deaths in the 12-month period ending in April 2021, an estimated 100,306, representing an increase of 28.5% over the previous 12 months [1]. These new estimates indicate a sharp departure from recent declines and are largely attributed to the COVID-19 pandemic [2]. The majority of these deaths involve opioids, which has claimed over 500,000 lives in the past two decades [3]. For every overdose death, thousands more have experienced nonfatal overdose, addiction, and morbidities such as abscesses and infectious diseases (e.g., HIV, HCV). In many jurisdictions, these sequelae are complicated and exacerbated by a policy environment which largely treats drug use as a criminal justice rather than public health issue. The criminalization of drugs and drug paraphernalia and its disparate application has erected barriers to accessing evidence-based harm reduction and public health prevention resources and exacted a large toll on communities of color, creating further health disparities and police mistrust [4].

Structural interventions are sorely needed given the nature and extent of the opioid epidemic in the USA. Overdose prevention sites (OPSs), a key feature of many public health responses to opioid misuse globally and has been proposed in a number of US jurisdictions with two OPS having opened at the close of 2021. OPS, also called safe consumption spaces or safe injection sites, are defined as spaces in which individuals consume previously purchased drugs under supervision [5]. There are over 120 OPS in 10 countries worldwide [6]. Evidence on the impacts of OPS demonstrates their significant association with reduced HIV [7] and HCV transmission [8], syringe sharing [9], public injection, and crime [10, 11] and has been found to be cost effective [12, 13]; OPSs have been found to be associated with reductions in overdose deaths even in surrounding neighborhoods in Canada [14] and the more recently USA [10] as well as reductions in neighborhood nuisances such as public injection and discarded syringes [15, 16].

OPSs remain legally prohibited in the USA despite indications of high support among studies of people who use drugs (PWUD) [17–20] as well as moderate support (45%) among a nationally representative sample [21]. There are no formally sanctioned OPS and one documented unsanctioned OPS in the USA [22]. Efforts to overcome legal barriers are ongoing in a number of locales, yet local opposition hampers implementation. Concerns largely center around two issues. First, OPSs are viewed as condoning and encouraging drug use rather than eliminating it [16, 23, 24]. Secondly, they are perceived as amplifiers of drug and crime-related problems in the neighborhoods in which they are located, a fear that has been refuted by public health evidence [11, 23, 25]. Such concerns are referred to as “NIMBYism (Not In My Back Yard),” which describes a community’s resistance to the placement of services such as drug treatment programs [26], syringe services programs (SSPs) [27, 28], and recently, OPS in their immediate community [29, 30].

Community opposition to drug treatment and SSPs are often led by local businesses who worry about the economic impact on their businesses. At the same time, businesses have much to gain by addressing neighboring drug use. Business owners, a key constituency for city and state legislators, could provide a powerful, untapped potential advocate to implement evidence-based policies, and harm reduction interventions. Characterizing business owners and employees’ awareness of existing harm reduction resources and support or concern about planned strategies is therefore central to enacting effective public health prevention of overdoses and fatalities. Here, we describe their experiences with drug use and overdose and examine the correlates of OPS acceptability among businesses located in areas with concentrated drug activity in an urban epicenter of the contemporary overdose epidemic.

## Methods

### Study Setting and Data Collection

We conducted a cross-sectional study of businesses in Baltimore, Maryland using two recruitment methods: in-person data collection occurred between December 2019 and March 2020; and telephone-based surveys were implemented between April and July 2020 during the COVID-19 pandemic. Recruitment locations (“zones”) were areas of geographically concentrated drug arrests identified by heat mapping 2018–2019 drug arrest location data using ArcGIS [31, 32]. We next conducted “windshield tours” of each of the 13 identified zones in June–July 2019 to record the name and address of every business within those regions. Windshield tours are drives throughout a given geographic area by study staff in which information is systematically recorded. This generated a list of eligible businesses (*N* = 891) for recruitment, categorized as retail, corporate food and beverage chain stores, liquor stores, corner stores, food/restaurants, barber shops/salons, and other businesses (i.e., library, self-storage company).

Next, we employed an iterative, two-phased sampling recruitment strategy. To ensure a minimum level of representation from each zone, the first data collection phase randomly recruited at least eight businesses per zone. In the second phase, we randomly selected a list of businesses to approach each week, without continuing restriction by zone, through a random number generator in Stata 15.1 (StataCorp, College Station, TX). During the first phase of recruitment, we visited 340 businesses and 33% of businesses agreed to study participation. Reasons for not participating included 27% were out of business by the time of data collection; 16% refused participation; and 12% were ineligible due to language barriers. The remaining 45% of businesses were screened out after more than three visit attempts but were not open, although in business. One hundred and twelve surveys were administered in person. During in-person data collection, staff pairs visited identified businesses and to gauge interest in study participation. If interested, surveys were administered after determining eligibility. Inclusion criteria for participating individuals were being an employee, manager, or owner of business for at least 6 months; working on the premises for at least 10 h a week; being 18 years of age or older; and being able to speak English. Informed consent was obtained orally from eligible participants, who were then administered a 25-min audio computer-assisted personal interview (ACASI). Upon completion, staffs were offered naloxone and provided with information sheets about what to do in the event of an overdose, where to replenish naloxone supply, and descriptions of ongoing OPS efforts in Baltimore.

Due to stay-at-home policies following COVID-19, we transitioned to a telephone-based survey in April 2020 to complete data collection. During this period, contact with the remaining eligible businesses (*n* = 551) was attempted through phone calls and social media (e.g., Facebook, Instagram) but 75% were unreachable due to phone numbers out of service (*n* = 128); and/or calls and messages were unanswered after at least five attempts (*n* = 288). Of the businesses that we were able to contact (*N* = 135), 27% agreed to participate (*N* = 98). If eligible and consented to participate, surveys were interviewer-administered and lasted 60 min. Survey items were informed by discussions with harm reduction organizations, key informant interviews with PWUD (*n* = 5) and business managers (*n* = 2), and the literature [33]. Participants were compensated with prepaid VISA gift cards, $15 USD for in-person interviews and $40 USD for those over the phone, with the increased rate compensating for the additional length of time to administer the survey.

We survey 149 participants at unique businesses across both recruitment phases. In this study, we included those who completed all questions on attitudes towards OPS. A sensitivity analysis found that individuals who did not complete these questions were significantly older than those who did not (mean age = 48 vs 40; *p* = 0.030), with no other significant differences in individual or business characteristics. Thus, the final analytic sample comprised of *N* = 135 survey respondents.

### Measures

The primary outcome, referred to as local support of OPS, was measured by assessing agreement with the statement: *I would be comfortable with an OPS in this neighborhood*. We also measured overall support of OPS, assessed by agreement with the following statement: *I would be comfortable with an OPS in a different neighborhood.*

Exposure variables were comprised of those at the individual level, including sociodemographic characteristics (e.g., age, gender, race, and education level), work-related characteristics including role (e.g., owner, manager, and employee), mean weekly number of work hours, mean number of years at the business, and if the participant lived near their work (yes/no). Role in business was collapsed into a binary indicator representing owners versus managers/employees. Business characteristics included business type (i.e., food, corner store, and retail), categories of the number of customers per day (< 15, 15–99, 100 +), bathroom policy (employee only vs. open to public), and employee drug testing policies (yes/no). Experiences with personal drug were ascertained by asking about whether participants had ever done drugs excluding marijuana (yes/no). Perceived frequency of PWUD visiting the workplace was measured by a dichotomized Likert-scale variable (rarely/never vs. sometimes/always).

Attitudes towards PWUD were measured by four-item scale comprised of the following statements with a 5-point Likert response pattern (strongly agree, somewhat agree, somewhat disagree, and strongly disagree): PWUD should be arrested (reversed), PWUD are dangerous (reversed), PWUD deserve respect, and PWUD deserve access to drug treatment. The alpha coefficient for this scale was moderately adequate, at 0.63, with higher scale scores indicating positive attitudes toward PWUD. We ascertained workplace exposures to drugs through several variables. Perceptions of commonly used drugs (e.g., heroin, cocaine, marijuana, crystal methamphetamine, and opioid pills) by PWUD around the workplace in the past 6 months were measured using binary (yes/no) responses. Drugs were then reduced into three categories (e.g., marijuana/synthetic cannabinoids, opioids, and stimulants). Workplace drug exposure was characterized by seven binary (yes/no) responses about: (1) finding drugs or paraphernalia inside the business; (2) finding drugs or paraphernalia around business; (3) seeing drug use or drug dealing inside the business; (4) seeing drug use or drug dealing around the business; (5) receiving staff or customer complaints about drug use; (6) asking people to leave or banning them due to drug use; and (7) calling 911 due to drug use in the past 6 months. Higher scores indicated increased exposure to drug use in the workplace, and the scale’s alpha coefficient was 0.85. Witnessing an overdose (yes/no) was considered separately, owing to its markedly higher level of associated trauma [34] compared to the other scale items.

Familiarity and experiences with naloxone were ascertained in a series of binary questions (yes/no) including having heard about naloxone, having naloxone in the workplace, having employees trained in naloxone administration, and having administered naloxone. We ascertained if participants had heard of an OPS (yes/no) prior to study participation, providing participants with a common definition as a point of reference to ensure consistency. Lastly, examined perceived impacts (yes/no) of OPS on a number of negative (e.g., enable drug use, attract PWUD, attract drug dealing, attract crime, and negatively impact their business) and positive outcomes (e.g., reduce drug deaths, reduce drug paraphernalia, and benefit the community).

### Analysis

Exposures of interested were stratified by the primary outcome of interest, local support of OPS. Bivariate *p*-values were calculated using Pearson’s *χ*2 tests for binary and categorical variables, Fisher’s exact tests where sample sizes were below 10 per category, or *t*-tests for continuous variables. Row percentages are displayed in Tables 1 and 2 while column percepts are reported in the results. A multivariable logistic regression model was constructed to model correlates of support of OPS in neighborhood of the respondent’s business, establishment with robust standard errors to account for clustering by zone of recruitment. Items with bivariate *p* < 0.20 were considered for inclusion in a multivariable model, with a few exceptions. Employee-only bathroom policy was excluded due to its relationship with business type (i.e., establishments such as libraries, or those serving food, were unable to implement such a policy, thus introducing bias). Type of drug used in the neighborhood was excluded due to small cell sizes and high levels of overlap between items. Personal drug use history and naloxone-related variables were excluded due to small sample sizes. Estimates were adjusted for hours per week due to the relationship between hours worked, role in business, and OPS support. Finally, perceived negative and positive impacts of OPS were described among those who supported OPS in the neighborhood of their business establishment, generally, or not at all. All analyses were conducted in Stata/SE 15.1 (College Station, Texas).

**Table 1:** Characteristics of CONNECT participants and the businesses in which they work stratified by OPS support in Baltimore, Maryland (*N* = 135)

	Total	Support OPS in their businesses neighborhood		*p*-value^*^
	(*N* = 135)	No (*n* = 47; 35%)	Yes (*n* = 88; 65%)	
	Col %	Row %	Row %	
Sociodemographic data				
Age	40.1 (12.5)	41.5 (12.7)	39.3 (12.5)	0.338
Gender				
Male	78 (57.8)	29 (37.2)	49 (62.8)	
Female	57 (42.2)	18 (31.6)	39 (68.4)	0.500
Race				
NH White	25 (18.5)	10 (40.0)	15 (60.0)	
NH Black	65 (48.1)	20 (30.8)	45 (69.2)	
Other	45 (33.3)	17 (37.8)	28 (62.2)	0.625
Education level				
High school/GED or less	54 (40.0)	17 (31.5)	37 (68.5)	
Some college or more	81 (60.0)	30 (37.0)	51 (63.0)	0.507
Personal drug use history				
Ever used illicit^+^ drugs (excluding marijuana)	23 (17.2)	7 (30.4)	16 (69.6)	0.811
Role in business				
Owner	33 (24.4)	14 (42.4)	19 (57.6)	
Manager or employee	102 (75.6)	33 (32.4)	69 (67.6)	0.291
Years at business	7.0 (8.9)	8.1 (11.1)	6.3 (7.4)	0.648
Hours worked per week	48.3 (15.4)	45.3 (16.2)	49.9 (14.7)	0.474
Live near work	49 (36.3)	12 (24.5)	37 (75.5)	0.057
Characteristics of business				
Avg number of customers served per day				
< 15	34 (26.6)	9 (26.5)	25 (73.5)	
15–99	48 (37.5)	14 (29.2)	34 (70.8)	
100 +	46 (35.9)	21 (45.7)	25 (54.3)	0.144
Employee-only bathroom policy	92 (69.7)	38 (41.3)	54 (58.7)	0.018
Drug testing is conducted among employees	25 (18.7)	11 (44.0)	14 (56.0)	0.259

^*+*^includes all routes of administration of heroin, crack/cocaine, cocaine, nonmedical use of prescription opioids

**Table 2:** CONNECT participants experience with and attitudes towards drug use, OPS, and other harm reduction interventions stratified by OPS support in Baltimore, Maryland (*N* = 135)

	Total	Support OPS in neighborhood		*p*-value
	(*N* = 135)	No (*n* = 47; 35%)	Yes (*n* = 88; 65%)	
	Col %	Row %	Row %	
Attitudes towards people who use drugs				
Should be arrested	60 (44.4)	26 (43.3)	34 (56.7)	0.063
Are dangerous	77 (57.0)	36 (46.8)	41 (53.2)	0.001
Deserve respect	107 (79.3)	37 (34.6)	70 (65.4)	0.911
Deserve access to treatment	129 (95.6)	42 (32.6)	87 (67.4)	0.011
Workplace exposures				
Some or all people entering the business use drugs	122 (91.7)	41 (33.6)	81 (66.4)	0.195
Perceived drugs commonly used around workplace^+^				
Opioids (fentanyl, heroin, pills)	107 (84.3)	30 (28.0)	77 (72.0)	0.018
Stimulants (cocaine, crack cocaine, crystal methamphetamine)	98 (77.2)	29 (29.6)	69 (70.4)	0.233
Workplace drug exposure scale components ^*+*^				
Found drugs	63 (48.1)	19 (30.2)	44 (69.8)	0.331
Found drug paraphernalia	65 (49.6)	21 (32.3)	44 (67.7)	0.625
Seen drug use	87 (66.4)	26 (29.9)	61 (70.1)	0.130
Seen drug dealing	90 (68.7)	30 (33.3)	60 (66.7)	0.716
Asked somebody to leave or banned them due to drug use	82 (61.2)	26 (31.7)	56 (68.3)	0.305
Called 911 due to drug use or overdose	46 (34.3)	15 (32.6)	31 (67.4)	0.665
Witnessed an overdose in or around business^+^	61 (46.6)	15 (24.6)	46 (75.4)	0.024
Exposure to harm reduction interventions				
Heard of naloxone	94 (69.6)	28 (29.8)	66 (70.2)	0.063
Had naloxone in business	35 (37.6)	8 (22.9)	27 (77.1)	0.255
Any employees trained to administer naloxone	44 (48.9)	10 (22.7)	34 (77.3)	0.171
Ever administered naloxone	10 (7.6)	2 (20.0)	8 (80.0)	0.493
Heard of OPS before this study	56 (41.5)	15 (26.8)	41 (73.2)	0.142

^*+*^past 6 months

## Results

A majority of participants (65%) supported OPS in the neighborhood in which their business was located (Table 1) while 85% supported OPS regardless of location (not shown). Participants were an average age of 40 years old, predominantly male (58%), Black (48%), and reported at least some college education (60%). Seventeen percent reported ever having used illicit drugs, excluding marijuana. Twenty-four percent reported being business owners, 36% managers, and 40% employees. Participants worked at their businesses for an average of 7 years, averaging 49 h a week of work. About one-third (36%) reported living near work. The most common business types in which participants worked were food service such as dine in/take out restaurants and bars with food (30%), retail businesses (23%), corner stores (13%), barber shop/salons (11%), and liquor stores (7%). Daily business volume varied widely with 27% reported less than 15 customers daily, 38% reported 15–99 customers, and 36% reported over 100 customers daily. Drug testing for employees was reported by 19% of the sample.

Experience with and attitudes toward drug use and PWUD are displayed in Table 2. Less than half (44%) of participants felt that PWUD should be arrested for drug use, and 57% believed that PWUD were dangerous. Yet, 79% felt PWUD “deserved respect,” and 96% felt PWUD should have access to treatment. Support for OPS locally was significantly lower among participants who believed PWUD were dangerous compared to those who did not support OPS locally (53% vs. 81%). Support for OPS locally was significantly higher among participants who believed PWUD deserve access to treatment (67% vs 17%) and reported that opioids were commonly used in or around their workplace (72% vs 45%) compared to those who did not support OPS locally.

Almost all participants (92%) perceived at least some of the people who entered their business were PWUD. Opioids (84%) and stimulants (77%) were perceived to be used extensively in the immediate area surrounding participants’ businesses. Exposure to drug use in the workplace was also common. Participants frequently found drugs (48%) or drug paraphernalia (50%) and witnessed drug use (66%) and drug dealing (69%) in or around their business. Almost half (47%) had witnessed an overdose in or around their business in the past 6 months, with a significantly higher percent of those who witnessed an overdose in or around their business in support of OPS locally (75% vs 57%). Almost two thirds (61%) of participants said that employees had to ask people to leave or banned them from the premises due to drugs. About a quarter of the sample had called 911 in response to either somebody being under the influence (26%) or experiencing an overdose (25%) in the past 6 months (34% total had called for either drug use or overdose). A vast majority (70%) heard of naloxone, and 38% reported having it on the premises. Close to half (49%) said that some employees were trained in how to administer naloxone, and 8% had personally done so in the past 6 months. Only 41% had previously heard of OPS.

Controlling for relevant variables, correlates of supporting an OPS locally (Table 3) are living in the same neighborhood as work (adjusted odds ratio (aOR) 1.99, 95% confidence intervals (CI): 1.30–3.05); having a more positive attitude towards PWUD (*aOR* 1.33, 95% *CI*: 1.13–1.58); and having recently seen an overdose in or around the workplace (*aOR* 2.86, 95% *CI*: 1.11–7.32). Lack of support for an OPS locally was being an owner, rather than a manager or employee, of the business (*aOR* 0.35, 95% *CI*: 0.15–0.83).

**Table 3:** Correlates of supporting OPS locally among CONNECT participants (*N* = 135) in Baltimore, Maryland

	Unadjusted estimates		Adjusted estimates	
	Odds ratio (uOR)	95% *CI*	Odds ratio (aOR)	95% *CI*
Individual characteristics				
Role in business				
Owner vs employee/manager	0.65	0.31–1.36	0.35	0.15–0.83
Live near work	2.11	1.00–2.13	1.99	1.30–3.05
Experiences with and attitudes towards PWUD				
Attitudes towards PWUD scale	1.22	1.04–1.45	1.33	1.13–1.58
Workplace exposure scale	1.10	0.96, 1.18	0.98	0.83–1.15
Seen overdose in/around workplace	2.78	1.52, 5.12	2.86	1.11–7.32

^*+*^Adjusted for hours worked per week

**Fig. 1:**
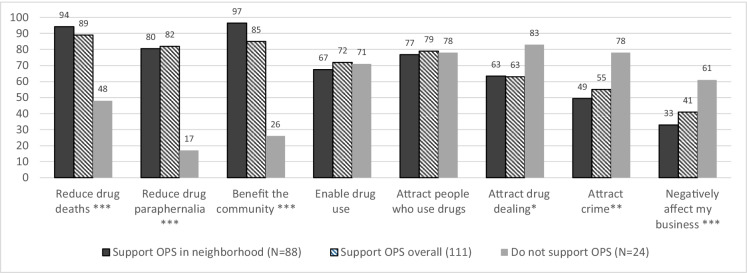
Perceived impacts of OPS among CONNECT participants (*N* = 135) in Baltimore, Maryland. *p < *0.1*; **p < *0.05*; ***p < *0.001*

Figure 1 exhibits the participants’ beliefs surrounding the impact of an OPS if implemented, stratified by whether they supported OPS locally, supported OPS regardless of geographic location, or not at all. Perceived benefits including the belief that OPS can reduce deaths, reduce drug paraphernalia, and confer an overall community benefit were significantly higher among those who supported OPS both locally and overall compared to non-supporters. Concerns about OPS attracting drug dealing, crime, and negatively impacting their business was significantly higher among those who did not support OPS compared to the other two categories.

## Discussion

We documented high levels of support for local OPS among a diversity of businesses situated in neighborhoods characterized by high levels of drug activity. Less than half of study particpants had previously heard of an OPS, signaling a need to raise awareness, conduct outreach, and deliver evidence-based public health messaging to this community specifically. However, once the concept was explained, most business owners and employees expressed support for an OPS in their neighborhood and an even greater number supported OPS generally. We found similar levels of local and broad OPS support to that of a study among business owners/managers in Kensington, Pennsylvania, where the opening of the first sanctioned OPS was delayed by legal barriers [33]. Our study indicates that OPS support and more empathetic attitudes towards PWUD were driven by personal experiences, with living near your workplace and having recently witnessed an overdose at work being significantly associated with OPS support in an adjusted model. Supporters felt that OPS they would reduce drug deaths and paraphernalia and broadly benefit the communities surrounding their businesses, while opponents expressed concern that OPS would attract crime, drug dealing, and negatively impact business. These findings can inform how to engage and build coaltions with businesses in OPS advocacy as well as inform OPS message framing aimed at the business community.

Results illustrate that business owners and employees are on the front lines of the opioid epidemic and as such are confronted with drug use sequelae daily. In some instances, these encounters resulted in customer complaints, asking customers to leave, or calling 911 in the event of an overdose. Engaging personal narratives (i.e., witnessing overdose) in advocacy efforts among business stakeholders, other community members, and elected officials could serve as a powerful tool of persuasion in the public policy making process [35, 36].

Lower support expressed by owners compared to employees could reflect the fear of a financial impact of an OPS outweighing perceived benefits to the neighborhood or needs of PWUD. Indeed, when we ascertained perceived impacts of OPS, concerns that OPS would attract drug dealing, crime, or negatively affect their business were significantly associated with reduced OPS support. Fears surrounding this “honeypot effect” of OPS have been repeatedly refuted in the literature [25, 29, 37, 38]. Research among business owners following the opening of Sydney’s OPS documented significant decreases in witnessed public injection and public discarded injection equipment in the prior month [38]. Nonetheless, these concerns remain challenging to dispel. Contributing factors to their persistence may include outsized faith in anecdotal evidence, stigma towards PWUD [39], the lack of familiarity with the existing evidence base, disbelief in science, or belief that the evidence, particularly from international settings cannot be generalized locally. In the USA, recent research on neighborhood effects of an unsanctioned safe consumption site noted broader neighborhood-level reductions in crime [10]. In partnership with community advocates, researchers should partner with advocates to generate and effectively disseminate more local evidence to address ongoing misconceptions.

Results from this study should be viewed in light of several limitations. The study sample is not representative of the entire business community for several reasons. First, we could not recruit participants who were unable to complete the survey in English given study staff language limitations; however, we encountered many non-English-speaking or limited English-speaking business owners, managers, and employees. This likely resulted in the underrepresentation of important perspectives from immigrant communities who run businesses or work in neighborhoods with high levels of drug use. Second, there was a high turnover of businesses observed in all but one of our recruitment zones; results may therefore underrepresent individuals with tenuous financial stability, or those who left neighborhoods due to the very experiences we sought to measure. Finally, the unprecedented public health measures adopted during the COVID-19 epidemic interrupted in-person recruitment; as a result, many businesses were closed, and those who were difficult to reach (e.g., had no functional contacts listed on search engine and social media platforms) may have had different characteristics than those we were able to contact.

Yet, the study has a number of lessons to inform future research and policy efforts. We found encouraging levels support for OPS in the business community once the concept was explained. Concerns about increased crime and threats to business viability were noted and need to be directly addressed in order to engage businesses in OPS advocacy efforts among fellow business as well as legislaters. Findings indicate the importance of including these stakeholders in informational messaging campaigns and engaging personal narratives and local evidence to dispel myths surrounding OPS, particularly that this evidence suggests beneficial neighborhood effects which would positively impact on business owners and workers alike. Businesses should be included in organizing efforts in early stages of advocacy and planning to ensure their concerns and needs are addressed and forge strong relationships with new allies in harm reduction efforts.
